# Monolithic UV‐Laser Programming of Photothermally Meta‐Morphing SMA Structures: Dual‐Encoded Kirigami Mechanics and Photonic Absorbance

**DOI:** 10.1002/advs.74930

**Published:** 2026-03-23

**Authors:** Hyunsoo Kim, Manmatha Mahato, Hunpyo Ju, Ryanda Enggar Anugrah Ardhi, Ji‐Seok Kim, Ashhad Kamal Taseer, Myung‐Joon Lee, Hyunjoon Yoo, Il‐Kwon Oh

**Affiliations:** ^1^ National Creative Research Initiative for Functionally Antagonistic Nano‐Engineering Department of Mechanical Engineering Korea Advanced Institute of Science and Technology (KAIST) Daejeon Republic of Korea; ^2^ Electronics and Telecommunications Research Institute (ETRI) 218 Daejeon Republic of Korea; ^3^ KAIST InnoCORE PRISM‐AI Center Korea Advanced Institute of Science and Technology (KAIST) Daejeon Republic of Korea; ^4^ Department of Mechanical Engineering Hanbat National University Daejeon Republic of Korea

**Keywords:** 3D morphing, haptic interfaces, laser‐induced oxidation, metamaterials, photothermal SMA actuators, shape displays, UV‐laser programming

## Abstract

Photothermal shape memory alloys (SMAs) offer a promising pathway toward wireless soft robotic systems, yet their practical implementation remains limited by reliance on heterogeneous coatings and multistep processing to achieve sufficient light absorption and programmed mechanical response. Here, we present a monolithic UV‐laser programming strategy that transforms flat NiTi sheets into photothermal SMA meta‐morphing structures, where kirigami mechanics and photonic absorbance are dual‐encoded within a single SMA metamaterial platform. UV‐laser micromachining defines the kirigami architecture to prescribe 3D morphing amplitude and force output, while laser‐induced oxidation creates micro–nano porous TiO_x_ layers that dramatically enhance near‐infrared absorbance without external coatings. This coupled mechanical–optical encoding provides deterministic control over deformation, heating rate, and temporal actuation dynamics. A comprehensive design map links geometric parameters to mechanical performance, and spatial patterning of oxidation levels enables spatiotemporally sequenced actuation under uniform illumination, demonstrating material‐level photonic logic. Leveraging these capabilities, we construct a multi‐channel 3D morphing and haptic display driven solely by optical inputs. This laser‐encoded platform establishes a scalable and manufacturing‐ready framework for architecting multifunctional SMA morphing systems, opening new opportunities for adaptive surfaces, interactive haptics, and photonic soft robotics.

## Graphical abstract

A monolithic manufacturing strategy is presented for photothermal shape memory alloy (SMA) meta‐morphing structures. Using a single‐step UV‐laser process, the mechanical compliance is programmed via kirigami patterning while optical absorbance is simultaneously tuned through surface oxidation. This dual‐programming approach enables wireless, battery‐free soft robotic systems capable of spatiotemporally controllable 3D morphing and tactile cues without additive coatings.

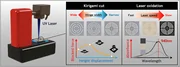

## 1 Introduction

Shape memory alloys (SMAs), particularly Nickel‐Titanium (NiTi), have garnered significant attention in the fields of soft robotics [1, 2, 3], artificial muscles [4, 5, 6], haptics [7, 8, 9], and deployable structures [10, 11, 12] due to their high energy density [13], large actuation strain [14], and biocompatibility [15]. Traditionally, SMA actuators are triggered via Joule heating, which necessitates tethered electrical connections and complex on‐board circuitry, thereby limiting the flexibility and scalability of soft standalone systems. To overcome these constraints, photothermal actuation has emerged as a powerful alternative, enabling wireless, remote‐controlled operation by converting optical energy into thermal stimuli. This approach allows for the development of untethered soft robots and reconfigurable metamaterials that can be selectively activated using spatial light modulators or laser beams [16, 17, 18, 19, 20].

Despite these advantages, the practical implementation of photothermally actuated SMAs faces a challenge related to the intrinsic optical properties of NiTi. Specifically, pristine Nitinol surfaces exhibit high reflectivity in the near‐infrared (NIR) region [21], resulting in poor photothermal conversion efficiency. The efficient photothermal actuation typically requires the application of external light‐absorbing layers onto the surface of the actuator. Common strategies include coating SMA surfaces with polymer‐based nanocomposites [21], electrochemical deposition of graphene oxide (GO) layers [22], or the utilization of structural inorganic films such as TiN, which can trap significant amounts of light [16].

However, these additive coatings present inherent functional and manufacturing limitations. Organic or physically deposited layers often exhibit poor adhesion and are susceptible to delamination under repeated thermomechanical cycling, requiring additional stabilizing structures or replacement with more complex inorganic films [16]. Moreover, the use of thick composite coatings to secure photothermal agents increases the thermal mass of the actuator, thereby reducing heating rates and response speeds [21]. Tailoring mechanical behavior and optical absorbance typically demands multiple disconnected fabrication steps—such as geometric shaping followed by chemical deposition or dip‐coating [22], which complicates processing and limits scalability. These challenges underscore the need for a unified manufacturing paradigm capable of deterministically encoding both the mechanical topology and optical surface properties of SMAs within a single monolithic material system. Such an approach must simplify workflow while delivering the precision, throughput, and scalability essential for the commercial deployment of soft robotic technologies [23].

In parallel with additive coatings, laser‐treated or surface‐modified NiTi has been investigated as a coating‐free route to tailor optical properties by forming micro–nano textures that suppress specular reflection and enhance light trapping. For example, femtosecond‐ and nanosecond‐laser structuring of NiTi has been reported to yield ultrabroadband absorption (“black metal”‐like behavior) and anti‐reflective and light‐trapping microstructures, where scan‐path‐dependent heat accumulation can promote porous textures beneficial for optical trapping [23, 24]. While these laser‐based studies demonstrate that surface texturing can improve absorption, they generally emphasize optical surface modification itself and do not address a unified, manufacturing‐ready framework that co‐programs the kirigami mechanical architecture with spatially patterned absorbance for multi‐channel, spatiotemporally programmed photothermal actuation in a single workflow.

Beyond coating‐based photothermal strategies, kirigami‐inspired actuators have emerged as a powerful route to achieve complex, reversible morphing by converting planar sheets into mechanically amplified 3D geometries. Recent studies have demonstrated highly programmable kirigami morphing in liquid crystal elastomer (LCE) sheets through topological patterns and profiles, enabling remotely programmed and reversible shape transformations [25], as well as light‐driven LCE kirigami with controlled molecular orientations for programmable actuation behaviors [26]. More recently, photoresponsive liquid crystal gel (LCG) kirigami has been extended to aquatic environments, demonstrating complex topological morphing and programmable motion in liquid media [27]. At the miniaturized end, kirigami‐style 2D‐to‐3D transformations have also been demonstrated at the nanoscale (nano‐kirigami), where in situ cutting and buckling of suspended metal nanofilms enable programmable 3D architectures and emergent optical responses (e.g., giant optical chirality) [28].

**FIGURE 1:**
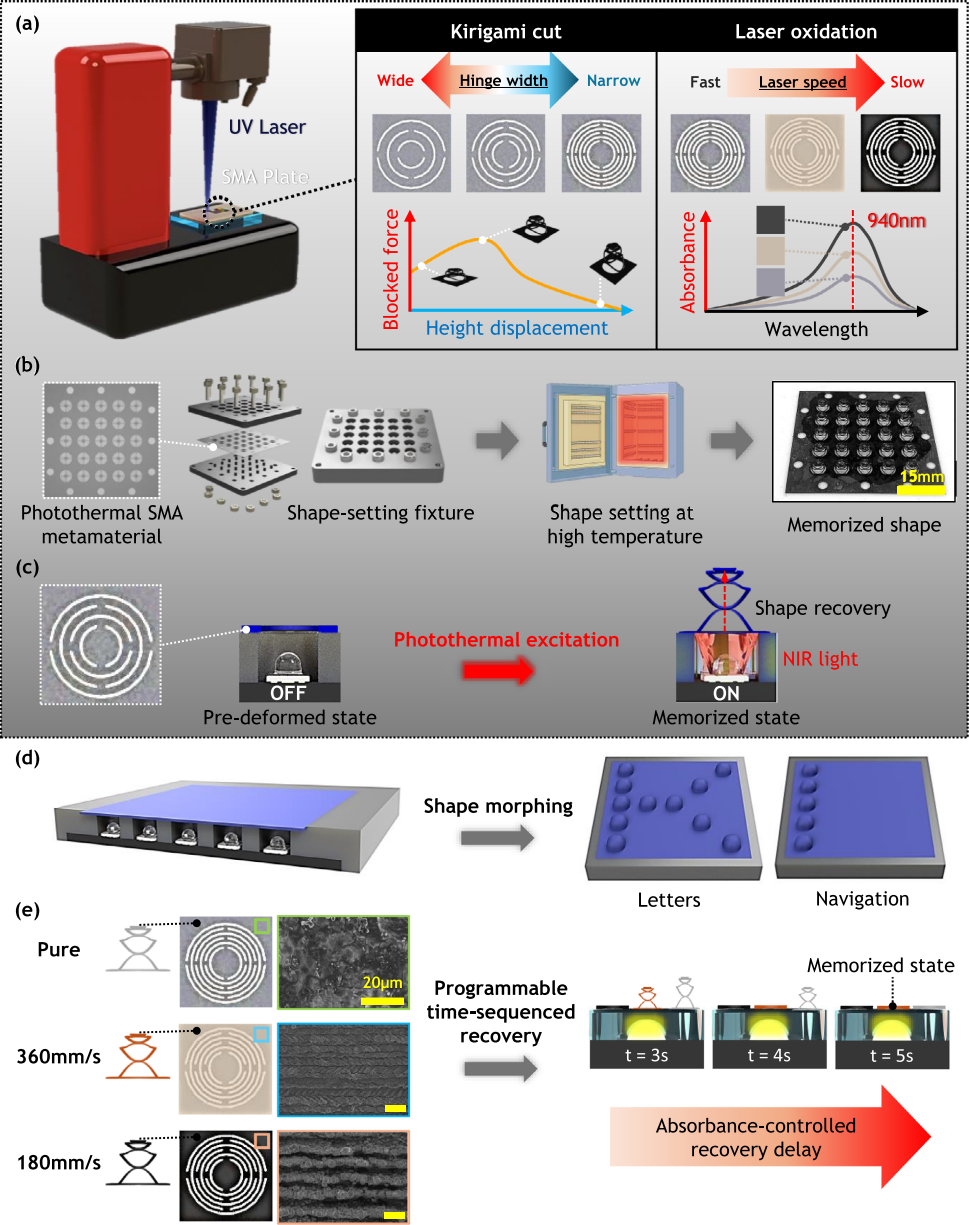
**Concept and monolithic fabrication strategy of the UV laser‐programmed photothermal SMA kirigami platform**. (a) Schematic illustrating the monolithic manufacturing concept. A single UV‐laser process enables dual‐functionality: geometric patterning (Kirigami cutting) to program mechanical outputs such as blocked force and height displacement, and surface oxidation to tune NIR optical absorbance. (b) Fabrication workflow of the actuator system, comprising UV‐laser micromachining of the SMA plate, mechanical shape‐setting using a dedicated fixture, and thermal treatment to imprint the memorized 3D geometry. (c) Working principle of the photothermal SMA actuator. Upon NIR illumination, the kirigami structure undergoes rapid heating and phase transformation, recovering from a pre‐deformed state to the memorized 3D configuration. (d) System‐level demonstration of a multi‐channel 3D morphing array integrated with an NIR LED matrix, exhibiting programmable applications such as letter formation and haptic navigation patterns. (e) Spatiotemporally programmed actuation via tailored laser oxidation. Distinct oxidation levels induce differential photothermal heating rates, enabling sequential shape recovery using a single light source.

While these advances highlight the versatility of kirigami architectures for shape programming in soft polymers and nanofilms, translating comparable levels of manufacturing‐ready, coating‐free photothermal programmability to macro‐scale SMAs remains challenging. This is largely because it requires simultaneous control of mechanical geometry and optical absorbance within a robust metallic platform. Motivated by these advances and remaining gaps, here, we introduce a monolithic manufacturing strategy to realize photothermal SMA meta‐morphing structures. Central to this strategy is the fabrication of a photothermal SMA metamaterial (PSM), in which kirigami mechanics and photonic absorbance are dual‐encoded within a robust metallic platform without additive coatings. As illustrated in Figure 1a, a single UV‐laser process simultaneously performs kirigami micromachining to program mechanical responses, such as blocked force and out‐of‐plane displacement, while inducing spatially controlled surface oxidation to tailor near‐infrared (NIR) absorbance. The streamlined fabrication workflow (Figure 1b) begins with UV‐laser micromachining of the NiTi sheet, followed by fixture‐assisted shape‐setting and thermal treatment to imprint the memorized 3D geometry. The resulting photothermal meta‐morphing unit (Figure 1c) rapidly heats and undergoes phase transformation under NIR illumination, recovering from a pre‐deformed state to its programmed 3D configuration. This monolithic platform readily supports system‐level integration (Figure 1d), enabling a multi‐channel 3D morphing array driven by an NIR‐LED matrix that renders programmable patterns such as letters and haptic navigation cues. Moreover, as shown in Figure 1e, patterning distinct oxidation levels across the structure encodes differential photothermal heating rates, yielding predictable, sequential shape recovery under a single uniform light source. Together, this approach establishes a robust framework for the co‐design and co‐encoding of mechanical and optical properties in intelligent soft actuators.

## 2 Results and Discussion

### 2.1 Geometric Programming of Kirigami Mechanics and Deformation Behavior

**FIGURE 2:**
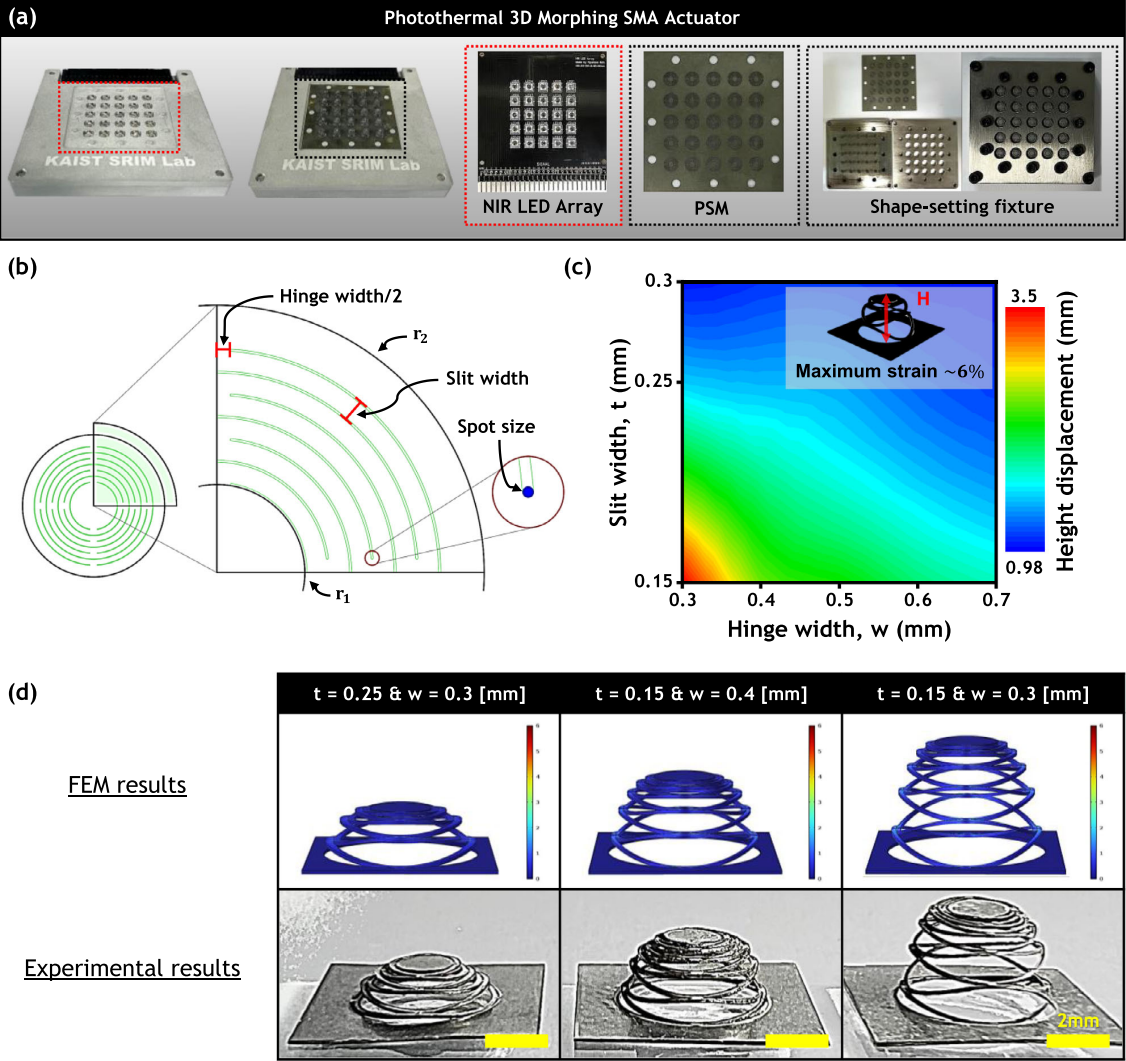
**Parametric geometric design and characterization of the photothermal SMA kirigami unit**. (a) Photographs of the integrated actuator assembly, showing the NIR LED array, the processed photothermal SMA metamaterial (PSM), and the shape‐setting fixture used for thermal training. (b) Geometric design parameters of the circular kirigami unit. The mechanical response is governed by the hinge width (w) and slit width (t). The inner/outer radii (r_1_ = 0.5 mm, r_2_ = 2.5 mm), UV‐laser spot size (25 µm), and plate thickness (T = 0.1 mm) were fixed for all experiments. (c) Finite Element Analysis (FEA) results presenting a contour map of vertical height displacement as a function of hinge width (w) and slit width (t), evaluated at a maximum principal strain of 6% within the structure. (d) Comparison between FEM‐predicted deformation profiles and experimental results for three representative kirigami geometries, demonstrating a strong correlation between the simulation and physical actuation behavior.

Kirigami was selected as the foundational geometry for the photothermal SMA morphing structures because it effectively converts a planar sheet into a mechanically amplified 3D pattern while preserving a large continuous surface area, which is critical for efficient NIR absorption. The physical realization of this concept is presented in Figure 2a, which displays photographs of the fully integrated actuator assembly comprising the NIR LED array, the PSM, and the shape‐setting fixture. Complementing this, Figure S1 details the specific assembly process, illustrating how the NIR‐LED PCB, the aluminum clamping frame, and the PSM are aligned and secured to form the final actuator.

In terms of geometric design, the architecture was optimized by minimizing the slit width (*t*) and tuning the hinge width (*w*) to sustain a high optical interaction area without compromising out‐of‐plane deformability. The circular kirigami unit is defined by fixed parameters such as inner and outer radii (r_1_ = 0.5 and r_2_ = 2.5 mm), SMA thickness (T = 0.1 mm), and UV‐laser spot size (25 µm), whereas *t* and *w* are varied to systematically control bending stiffness and attainable height displacement (Figure 2b). To predict the deformation behavior, finite element simulations were conducted to map vertical displacement as a function of (*t*, *w*) at maximum principal strain of ∼6%, which corresponds to the safe strain limit for NiTi SMA (Figure 2c). Because the fatigue life of NiTi SMAs is dictated by this maximum principal strain—ranging from low‐cycle fatigue (1–100 cycles) at a 6% strain limit to over 100 000 cycles at a ∼2% strain limit [29]—additional parametric maps were generated. Figure S2 and Table S1 detail the achievable morphing heights when the maximum principal strain is strictly constrained to 2%, 3%, 5%, and 6%. This provides a comprehensive design guideline for tailoring the kirigami geometry to balance desired morphing amplitude with specific fatigue‐life requirements. The comprehensive set of COMSOL‐predicted deformation profiles for all (*t*, *w*) combinations at a maximum principal strain of ∼6% is illustrated in Figure S3. Subsequently, the simulated actuation heights were utilized to define the pillar heights of the SUS 316/316L shape‐setting fixture, ensuring that each kirigami unit is thermally programmed to its specific target 3D geometry. As shown in Figure 2d, the close agreement between simulated and experimentally recovered shapes confirms that the deformation behavior of the kirigami units can be deterministically encoded through precise geometric selection and fixture‐assisted shape setting.

### 2.2 Mechanical Performance Mapping and Multi‐Channel System Integration

**FIGURE 3:**
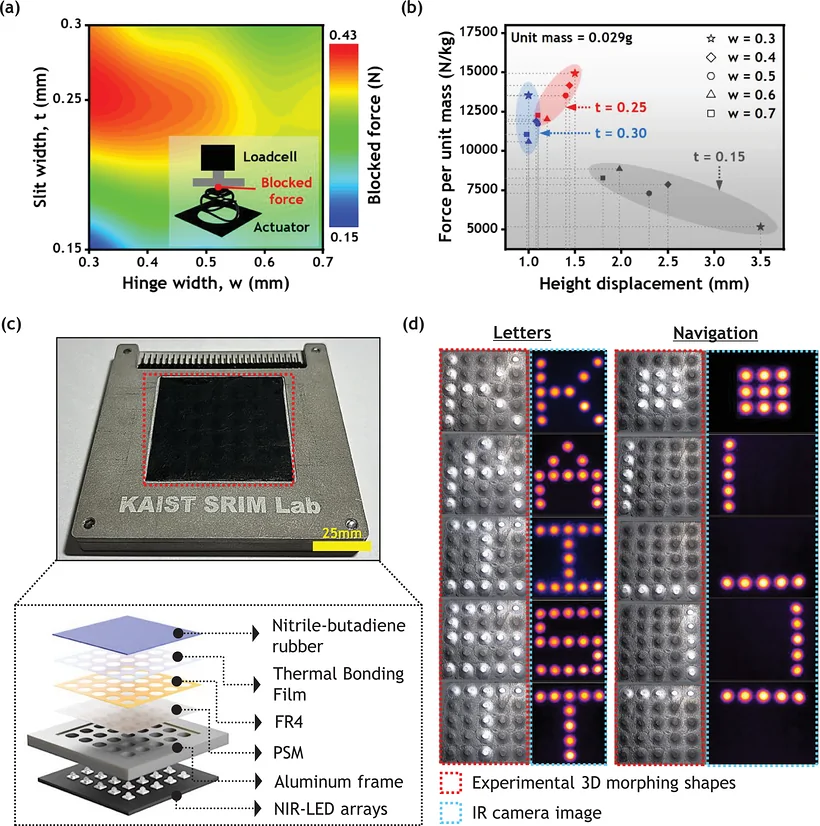
**Mechanical performance mapping and system‐level integration**. (a) Experimentally measured blocked force contour map obtained using a Universal Testing Machine (UTM). The force output is characterized as a function of hinge width (w) and slit width (t), quantifying the geometric dependence of the actuator's mechanical stiffness. (b) Performance trade‐off analysis plotting force‐per‐unit‐mass (specific force) versus height displacement for various kirigami designs. This map highlights the influence of geometric parameters (w and t) on mass‐normalized force generation and actuation amplitude. (c) Exploded view of the multi‐channel 3D SMA morphing actuator, detailing the functional layers: NIR‐LED array, SMA kirigami plate, FR4 substrate, thermal bonding film, and elastomeric encapsulation. (d) Multi‐channel demonstration of programmable 3D morphing. The array enables reconfigurable surface morphologies by selectively actuating SMA kirigami units under NIR illumination. Two demonstrations are shown: Letters (left group) and Navigation (right group). In the Letters demonstration, the array is sequentially morphed to render K → A → I → S → T. In the Navigation demonstration, it sequentially displays Stop → Left → Down → Right → Up to provide tactile guidance cues based on normal indentation (blocked‐force‐based contact). For each state, the actual out‐of‐plane 3D‐morphed configuration (enclosed by red dashed boxes) is shown on the left, alongside the corresponding IR‐camera view (enclosed by blue dashed boxes) on the right. The IR images visualize the spatially addressed NIR‐LED activation pattern (i.e., activated pixels) driving the associated PSM units. Because the out‐of‐plane morphing is less discernible in front‐view still images, the morphed units are visually highlighted for clarity. After each 3D morphing state, the array is manually reset to the pre‐deformed state before proceeding to the next pattern. The kirigami geometry used for this demonstration is w = 0.3 mm and t = 0.25 mm.

To establish comprehensive geometry–performance guidelines, the mechanical output of the PSM kirigami units was quantified via blocked‐force measurements using a universal testing machine with constrained vertical displacement (Figure 3a). The resulting contour map reveals a non‐monotonic dependence on slit width (*t*) and hinge width (*w*), indicating that peak blocked force emerges from intermediate geometries that optimally balance structural rigidity with compliant buckling. Furthermore, mass‐normalized performance is summarized in a trade‐off plot of specific force versus measured height displacement (Figure 3b), while the complete dataset comprising height, maximum blocked force, and force‐per‐unit‐mass for all (*t*, *w*) pairs is listed in Table S2. Within this design space, specific geometries such as (*t* = 0.25 mm, *w* = 0.3 mm) are identified as favorable for high‐force applications like tactile (cutaneous) stimulation, whereas designs like (*t* = 0.15 mm, *w* = 0.3 mm) are better suited for functions requiring large displacements, such as shape displays.

At the system level, the PSM is integrated with a multi‐channel NIR‐LED array and fixed using an aluminum frame (Figure 3c). This configuration provides independently addressable optical inputs to each kirigami unit directly on the SMA sheet without electrical wiring. Additionally, a soft elastomeric encapsulation layer was employed to act as both a thermal barrier and a contact‐smoothing interface to ensure safe skin interaction. The capability for multi‐channel actuation is demonstrated in Figure 3d, where letter patterns (Videos S1 and S2) and haptic navigation cues (Video S3) were selectively rendered by illuminating specific subsets of the kirigami units. Corresponding infrared images confirm localized heating at the activated sites, demonstrating that the kirigami SMA units successfully combine tunable mechanical performance with seamless integration into a scalable, optically addressable morphing platform. Note that these demonstrations operate in an open‐loop manner without integrated sensors to showcase the multi‐channel actuation capability of the 3D morphing platform.

### 2.3 Laser‐Induced Optical Tuning and Spatiotemporally Programmed Photothermal Actuation

**FIGURE 4:**
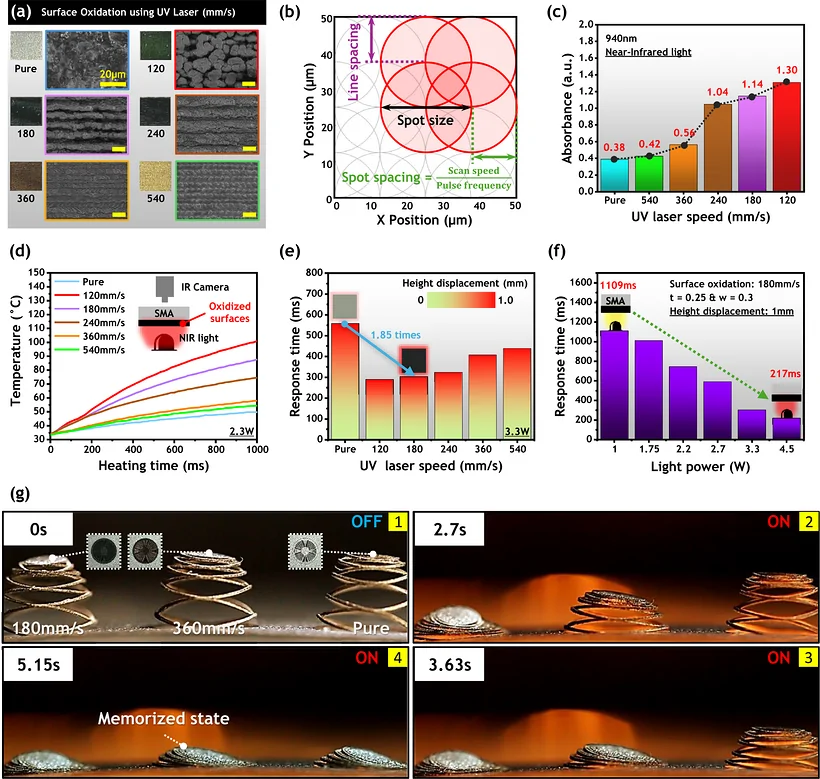
**Tunable optical properties and programmed temporal response via laser surface oxidation**. (a) SEM images showing UV‐laser‐induced surface oxidation at different laser scanning speeds. Lower laser speeds (longer dwell time) result in rougher and more porous surface morphologies. (b) Schematic of the laser oxidation conditions used in this study. A constant UV‐laser repetition rate of 60 kHz (spot size: 25 µm) was used, while the laser scanning speed and line spacing were adjusted to achieve uniform rastered oxidation across the SMA surface. (c) NIR absorbance as a function of laser scanning speed. Reduced laser speed increases the local energy input per unit area, producing stronger oxidation and consequently higher NIR absorption. (d) Photothermal heating profiles of oxidized SMA surfaces under identical NIR illumination, measured using an IR camera. The temperature rise rate correlates directly with the oxidation‐induced absorption contrast. (e) Response time required for the PSM to reach a height displacement of 1 mm under NIR actuation. Increased absorbance (achieved via lower laser speed) enhances photothermal heating efficiency, resulting in faster actuation response. (f) Response time measured at different NIR LED power levels for an SMA sample oxidized at 180 mm/s. A displacement of 1 mm is achieved within 217 ms at a maximum input power of 4.5 W. (g) Time‐sequenced recovery demonstration of spatiotemporal programming under a uniform optical input using three SMA samples (pure, 360 mm/s, and 180 mm/s oxidation). Unlike the near‐flat to popping‐up actuation driven by targeted NIR‐LEDs in (c–f), this specific experiment utilizes a single 50 W continuous broadband tungsten‐halogen lamp to simultaneously illuminate the units. Here, the structures exhibit a recovery from a popping‐up (pre‐deformed) state to a flat (memorized) state. Due to their distinct absorbance levels, the samples recover in a predictable sequence. This different memorized state was intentionally employed to demonstrate that the recovery kinetics consistently follow the programmed absorbance trends regardless of the mechanical shape‐memory state or the optical source type.

UV‐laser‐induced surface oxidation was employed as a materials‐level programming strategy to simultaneously tune optical absorbance and photothermal actuation dynamics. In particular, decreasing the laser scanning speed increases the local dwell time, thereby increasing the delivered energy and heat accumulation per unit area at the same location, producing rougher and more porous morphologies on the PSM surface. The porous texture enhances photon trapping and reduces specular reflection, thereby significantly increasing the NIR absorbance (Figure 4a,c), a finding consistent with the reported correlations between micro–nano roughness and photothermal conversion efficiency [30, 31]. Notably, the pure (non‐oxidized) SMA exhibits the lowest absorbance because its relatively smooth surface yields higher specular reflectance in the NIR, which limits photothermal conversion compared with the oxidized surfaces. Visual comparisons of laser‐processed SMA surfaces at various scan speeds, along with a detailed processing table specifying power, scan speed, pulse repetition rate, line spacing, and Z‐focus, are summarized in Figures S4b,c. The raster‐scanning scheme determining spot and line spacing was further clarified in Figure S4a in direct relation to the oxidation conditions (Figure 4b). The oxidation protocol utilizes a fixed repetition rate of 60 kHz and a 25 µm spot size, with scanning speed and line spacing adjusted to precisely control oxidation depth and surface texture.

This enhanced absorbance translates directly into a faster temperature rise under identical NIR illumination (Figure 4d), which subsequently shortens the actuation time required to reach a target morphing height of 1 mm (Figure 4e). Consequently, higher‐absorbance conditions enable the same deformation output with lower optical energy input, effectively improving actuation energy efficiency. To demonstrate the rapid actuation capability enabled by this efficiency, a representative geometry (*t* = 0.25 mm, *w* = 0.3 mm) oxidized at 180 mm/s was systematically characterized; as shown in Figure 4f, this optimized unit achieves a 1 mm displacement within ∼217 ms at an input power of 4.5 W. Full UV–VIS–NIR absorbance spectra (200–1400 nm) for all oxidation conditions are presented in Figure S5. While UV‐laser oxidation enhances photothermal heating, the cooling dynamics exhibit a non‐monotonic trend, revealing a decoupling between photon trapping and thermal dissipation (Figure S6). In passive cooling, pure SMAs cool slightly faster due to the absence of an oxide layer, whereas deep porous TiO_x_ structures (e.g., 180 mm/s) act as a modest thermal barrier [32]. Under active cooling, moderate surface roughness (e.g., 360 mm/s) slightly increases the convective contact area, whereas deeply porous structures (180 mm/s) can form localized “dead zones” that marginally degrade convective heat transfer [33]. Overall, the differences in cooling times across various oxidation conditions are relatively modest. Consequently, the choice of oxidation parameters primarily determines the photothermal heating efficiency, while the cooling behavior remains broadly comparable across the treated samples.

Finally, temporal programming was demonstrated in Figure 4g (Video S4), where pure, 360 mm/s‐oxidized, and 180 mm/s‐oxidized samples were illuminated simultaneously by a single continuous broadband tungsten‐halogen lamp. Despite the uniform input, they recover in a predictable sequence (180 mm/s → 360 mm/s → pure) dictated by their oxidation‐dependent light absorbency. For this specific demonstration, the units were intentionally programmed to achieve a popping‐up to near‐flat actuation. This approach explicitly demonstrates that the absorbance‐driven recovery kinetics operate independently of both the mechanical shape‐setting direction and the optical source type, proving the broad versatility of the dual‐encoding strategy. This visualization confirms that spatiotemporal logic can be embedded directly into the material through controlled and patterned oxidation, enabling photonic switching and time‐coded morphing without additional electrical addressability. Thus, UV‐laser oxidation emerges as a powerful route to co‐design NIR light absorbency, photothermal efficiency, actuation speed, and temporal sequencing in SMA kirigami metamaterials.

## 3 Conclusions

In this work, we introduce a monolithic UV‐laser programming strategy that transforms planar NiTi sheets into SMA meta‐morphing structures, where both kirigami mechanics and photonic absorbance are dual‐encoded within a single material system. Through UV‐laser micromachining, the mechanical architecture of the kirigami units is precisely defined to program deformation amplitude, blocked force, and 3D morphing capacity. Concurrently, laser‐induced oxidation generates micro–nano porous TiO_x_ layers that dramatically enhance NIR absorbance, enabling rapid, energy‐efficient photothermal actuation without any external coatings. The integrated geometric and optical encoding establishes a comprehensive design space that links hinge width, slit width, out‐of‐plane displacement, and mass‐normalized force output, providing quantitative guidelines for tailoring actuator performance. By spatially patterning oxidation levels, we further encode photonic logic into the material itself, enabling predictable, sequential actuation under uniform illumination. This materials‐level spatiotemporal programming demonstrates a fundamentally new route for achieving timing control, multi‐stage recovery, and photonic switching without electrical multiplexing. At the system level, the dual‐encoded SMA platform is seamlessly integrated with a multi‐channel NIR‐LED array to realize reconfigurable 3D morphing and tactile stimulation patterns, highlighting its scalability and application relevance. The combined mechanical–optical programmability establishes a unified paradigm for architecting functional soft morphing systems in a coating‐free, manufacturing‐ready manner.

Overall, this work advances SMA actuation beyond conventional photothermal or kirigami approaches by introducing a laser‐encoded meta‐morphing architecture in which mechanics, absorbance, and temporal behavior are co‐designed within a single processing framework. This paradigm opens new opportunities for adaptive surfaces, interactive haptics, photonic soft robotics, and next‐generation intelligent morphing interfaces where functional logic is embedded directly into the material.

## 4 Experimental Section

### 4.1 Materials and Instruments

Elastomeric encapsulation layers cut from laboratory‐grade nitrile–butadiene rubber (NBR) gloves (∼0.13 mm thick), 0.4‐mm‐thick FR4 substrates, NIR LEDs (λ = 940 nm; SST‐10‐IRD‐B50H‐U940, Luminus Devices), and thermoplastic adhesive films (3M Thermal Bonding Film 583, 50.8 µm) were commercially sourced and assembled to construct the multi‐channel 3D SMA morphing actuator. A custom SMA shape‐setting fixture was CNC‐machined from SUS 316L stainless steel. For global illumination and temporal programming demonstrations, a 50 W broadband tungsten‐halogen modeling lamp (GoodsGoods) mounted in a strobe housing (KoLight DX‐400) was utilized as a continuous VIS–NIR optical source. Surface temperatures during photothermal actuation were acquired using an infrared thermal camera (FLIR A35SC) and processed with Thermal Studio software, whereas blocked force was measured using a universal testing machine (AGS‐X, Dong‐il SHIMADZU). For the blocked force measurements, all shape‐set 3D SMA kirigami samples were initially pre‐deformed into a completely flat configuration on the testing stage. The universal testing machine tip was rigidly fixed at a zero‐displacement position to mechanically constrain any out‐of‐plane morphing. The photothermal recovery force was then recorded while maintaining this strictly flat state. Out‐of‐plane displacement was quantified with a laser triangulation sensor (optoNCDT 1420‐200, Micro‐Epsilon). NIR absorbance spectra were collected using a UV–VIS–NIR spectrophotometer (Lambda 1050, PerkinElmer), and laser‐oxidized surface morphologies were characterized via field‐emission SEM (SU8600, Hitachi High‐Tech).

### 4.2 Fabrication of the Photothermal SMA Metamaterial

Photothermal SMA metamaterials were fabricated from 0.1‐mm‐thick NiTi plates (austenite finish temperature ≈ 60 °C; Nexmetal Corporation), which were temporarily bonded to a glass substrate using a thin layer of glue‐stick adhesive to suppress vibration and prevent thermally induced warping during laser processing. Kirigami micromachining was carried out using a 3‐axis UV laser marker (Keyence MD‐U1000 series; spot size ≈ 25 µm) operated in cutting mode at 100% power, a scanning speed of 100 mm s^−^^1^, and a pulse frequency of 40 kHz; the cutting path was repeated 100 times with shallow ablation per pass to avoid excessive heat accumulation and ensure clean feature definition. Laser‐induced oxidation was subsequently performed using the engraving mode of the same system under fixed power (100%) and pulse frequency (60 kHz), while the scan speed and line spacing were varied to tune dwell time, oxidation depth, and resulting NIR absorbance.

### 4.3 Finite Element Method (FEM) Simulation

Finite element analysis was performed in COMSOL Multiphysics v6.0 using the Shell interface, where the kirigami geometry was imported from DXF and modeled as a 0.1‐mm‐thick NiTi sheet with isotropic linear elastic properties (ρ = 6400 kg/m^3^, E = 34 GPa, ν = 0.33). The outer frame edges were fully fixed to emulate clamping, and a prescribed out‐of‐plane displacement was applied to the innermost ring of the kirigami structure while enabling geometric nonlinearity. An auxiliary sweep function was used to incrementally increase the imposed displacement, and the resulting equilibrium shapes were solved using a stationary solver with locally refined triangular shell elements. Maximum principal strain (evaluated via the “shell.ep1” variable) was evaluated through a boundary maximum operator, and the actuation height corresponding to maximum principal strain of ∼6% was extracted for each (*t*, *w*) design pair to provide the displacement map in Figure 2c and determine the pillar heights for the SUS 316L shape‐setting fixture. Additionally, to establish geometric design guidelines corresponding to specific fatigue‐life limits, parametric sweeps were also conducted to systematically extract the achievable morphing heights at strictly constrained maximum principal strains of 2%, 3%, and 5% (detailed in Figure S2 and Table S1). Thermomechanical SMA phase‐transformation physics was not explicitly included because the purpose of this analysis was not to predict full thermomechanical actuation behavior but simply to estimate the geometric deformation envelope required for shape‐setting and fatigue‐life structural design; thus, a linear elastic approximation was sufficient for determining the fixture geometry.

## Author Contributions

H.K. performed the design, computations, fabrication, experiment, and demo. H.K. and M.M. wrote the manuscript, performed conceptualization, and performed the structural analysis. H.J., R.E.A.A., J.S.K., A.K.T., M.J.L., and H.Y. helped with figure draft and revision and contributed to configuring demonstrations. I.‐K.O supervised and managed the collaborations, revision, and writing of the manuscript. All authors discussed the results and commented on the paper.

## Conflicts of Interest

The authors declare no conflicts of interest.

## Supporting information




**Supporting File 1**: advs74930‐sup‐0001‐SuppMat.pdf

**Supporting File 2**: advs74930‐sup‐0002‐Video S1.mp4

**Supporting File 3**: advs74930‐sup‐0003‐Video S2.mp4

**Supporting File 4**: advs74930‐sup‐0004‐Video S3.mp4

**Supporting File 5**: advs74930‐sup‐0005‐Video S4.mp4

## Data Availability

The data that support the findings of this study are available from the corresponding author upon reasonable request.
