# Supplementary material for: Monolithic UV‐Laser Programming of Photothermally Meta‐Morphing SMA Structures: Dual‐Encoded Kirigami Mechanics and Photonic Absorbance
Source: Adv Sci (Weinh). 2026 Mar 23;13(31):e74930. doi: 10.1002/advs.74930 (PMC13587970; doi:10.1002/advs.74930)
Supplement: Supplementary file 1 [file ADVS-13-e74930-s002.pdf]

Supporting Information

Monolithic UV-Laser Programming of Photothermally Meta-Morphing SMA Structures: Dual-Encoded Kirigami Mechanics and Photonic Absorbance

Hyunsoo Kim[1], *Manmatha Mahato*[1], *Hunpyo Ju*[2], *Ryanda Enggar Anugrah Ardhi*[3], *Ji-Seok Kim*[1,4], *Ashhad Kamal Taseer*[1], *Myung-Joon Lee*[1], *Hyunjoon Yoo*[1] *and Il-Kwon Oh*[*,1,3]

H. Kim, M. Mahato, J.-S. Kim, A.K. Taseer, M.-J. Lee, H. Yoo and I.-K. Oh

[1]National Creative Research Initiative for Functionally Antagonistic Nano-Engineering, Department of Mechanical Engineering, Korea Advanced Institute of Science and Technology (KAIST), 291 Daehak-ro, Yuseong-gu, Daejeon 34141, Republic of Korea

H. Ju

[2]Electronics and Telecommunications Research Institute (ETRI) 218, Gajeong-ro, Yuseong-gu, Daejeon 34129, Republic of Korea,

R.E.A. Ardhi

[3]KAIST InnoCORE PRISM-AI Center, Korea Advanced Institute of Science and Technology (KAIST), Daejeon 34141, Republic of Korea

J.-S. Kim

[4]Department of Mechanical Engineering, Hanbat National University Daejeon 34158, Republic of Korea

[*] Correspondence and requests for materials should be addressed to I.-K. Oh (email: ikoh@kaist.ac.kr).

Keywords: photothermal SMA actuators; metamaterials; uv-laser programming; laser-induced oxidation; 3d morphing; haptic interfaces; shape displays

Table S1. Comprehensive mapping of achievable morphing height displacements for various combinations of hinge width (*w*) and slit width (*t*). The geometric designs are categorized by strictly constrained maximum principal strain limits (2%, 3%, 5%, and 6%) to guide fatigue-life design. The expected actuation cycles corresponding to each maximum strain limit are estimated based on established NiTi SMA fatigue literature.[29]

Cycle [29]		Above 100,000	Up to 100,000	Up to 100	1 ~ 100
Maximum strain		2%	3%	5%	6%
Hinge width, w (mm)	Slit width, t (mm)	Height displacement (mm)	Height displacement (mm)	Height displacement (mm)	Height displacement (mm)
0.3	0.15	1.02	1.58	2.79	3.5
0.3	0.25	0.46	0.7	1.22	1.5
0.3	0.3	0.31	0.48	0.83	1
0.4	0.15	0.78	1.2	2.11	2.5
0.4	0.25	0.43	0.67	1.19	1.44
0.4	0.3	0.34	0.51	0.89	1.08
0.5	0.15	0.68	1.05	1.85	2.3
0.5	0.25	0.41	0.65	1.15	1.4
0.5	0.3	0.34	0.53	0.93	1.1
0.6	0.15	0.6	0.93	1.62	1.98
0.6	0.25	0.35	0.55	0.98	1.2
0.6	0.3	0.3	0.48	0.85	1
0.7	0.15	0.54	0.84	1.45	1.8
0.7	0.25	0.32	0.5	0.88	1.1
0.7	0.3	0.29	0.45	0.8	0.98

Table S2. Mechanical performance data of photothermal SMA kirigami units. Height displacement, maximum blocked force, and force-per-unit-mass values are listed for all combinations of slit width (*t*) and hinge width (*w*), providing the quantitative dataset used to construct the mechanical performance trade-off map in Figure 3b of the main text.

t (mm)	w (mm)	Height (mm)	Maximum block force (N)	Force per unit mass (N/kg)
**0.15**	**0.30**	**3.5**	**0.14996**	**5171.034483**
0.15	0.40	2.5	0.22821	7869.310345
0.15	0.50	2.3	0.21192	7307.586207
0.15	0.60	1.98	0.25682	8855.862069
0.15	0.70	1.8	0.24002	8276.551724
**0.25**	**0.30**	**1.5**	**0.43276**	**14922.75862**
0.25	0.40	1.44	0.41061	14158.96552
0.25	0.50	1.4	0.39203	13518.27586
0.25	0.60	1.2	0.34842	12014.48276
0.25	0.70	1.1	0.35578	12268.27586
0.30	0.30	1	0.39225	13525.86207
0.30	0.40	1.08	0.34468	11885.51724
0.30	0.50	1.1	0.33977	11716.2069
0.30	0.60	1	0.30711	10590
0.30	0.70	0.98	0.32049	11051.37931

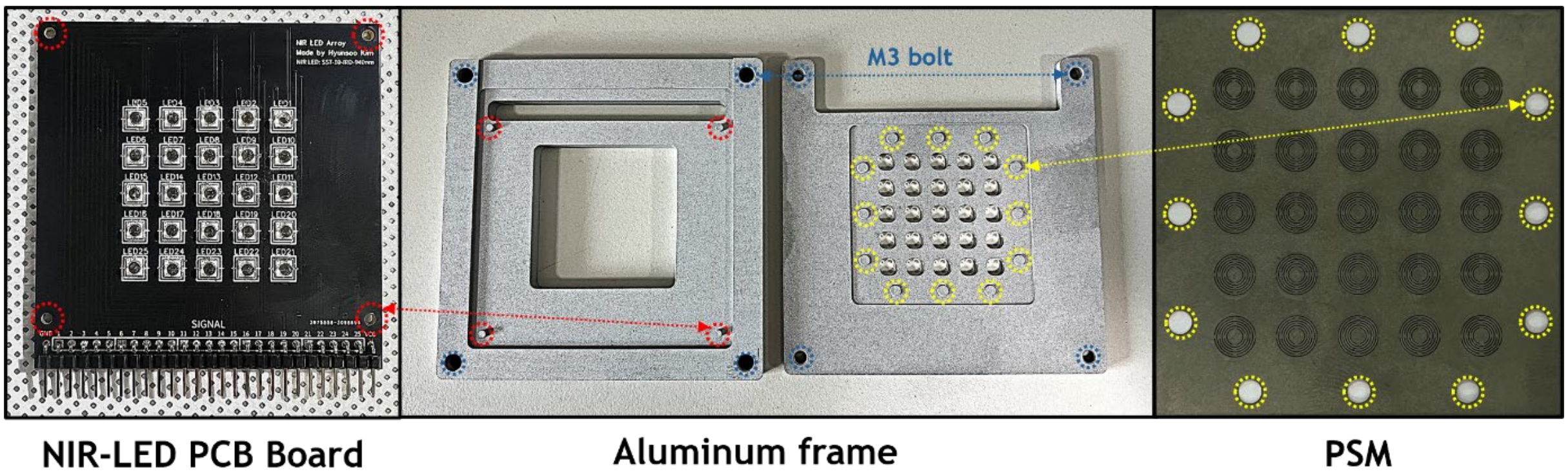

Figure S1. Assembly details of the multi-channel photothermal 3D morphing SMA actuator. The NIR-LED PCB board, aluminum frame secured with M3 bolts, and the photothermal SMA metamaterial (PSM) are shown together to illustrate the complete actuator assembly and the alignment of the kirigami plate with the LED illumination region.

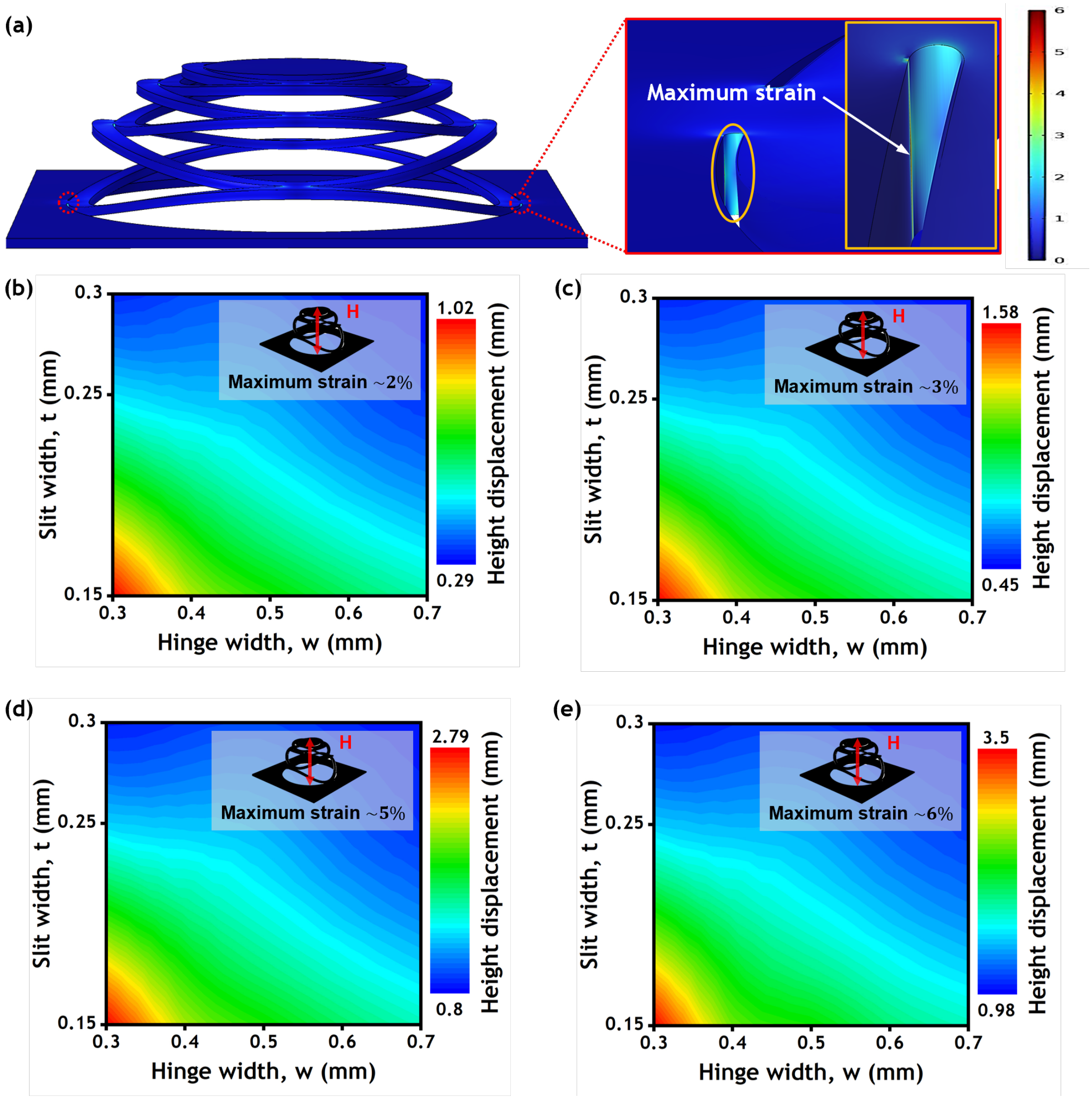

Figure S2. COMSOL-predicted deformation profiles and parametric height displacement maps constrained by maximum principal strain. a) Simulated 3D morphing profile with an inset highlighting the significant strain concentration highly localized at the hinge extremities (slit tips), which is a fundamental characteristic of the kirigami architecture enabling large out-of-plane deformation. b–e) Contour maps of the achievable vertical height displacement as a function of hinge width (*w*) and slit width (*t*), evaluated at strictly constrained maximum principal strain limits of 2%, 3%, 5%, and 6%, respectively. These maps provide a comprehensive visual guideline for tailoring morphing amplitude against specific fatigue-life requirements.

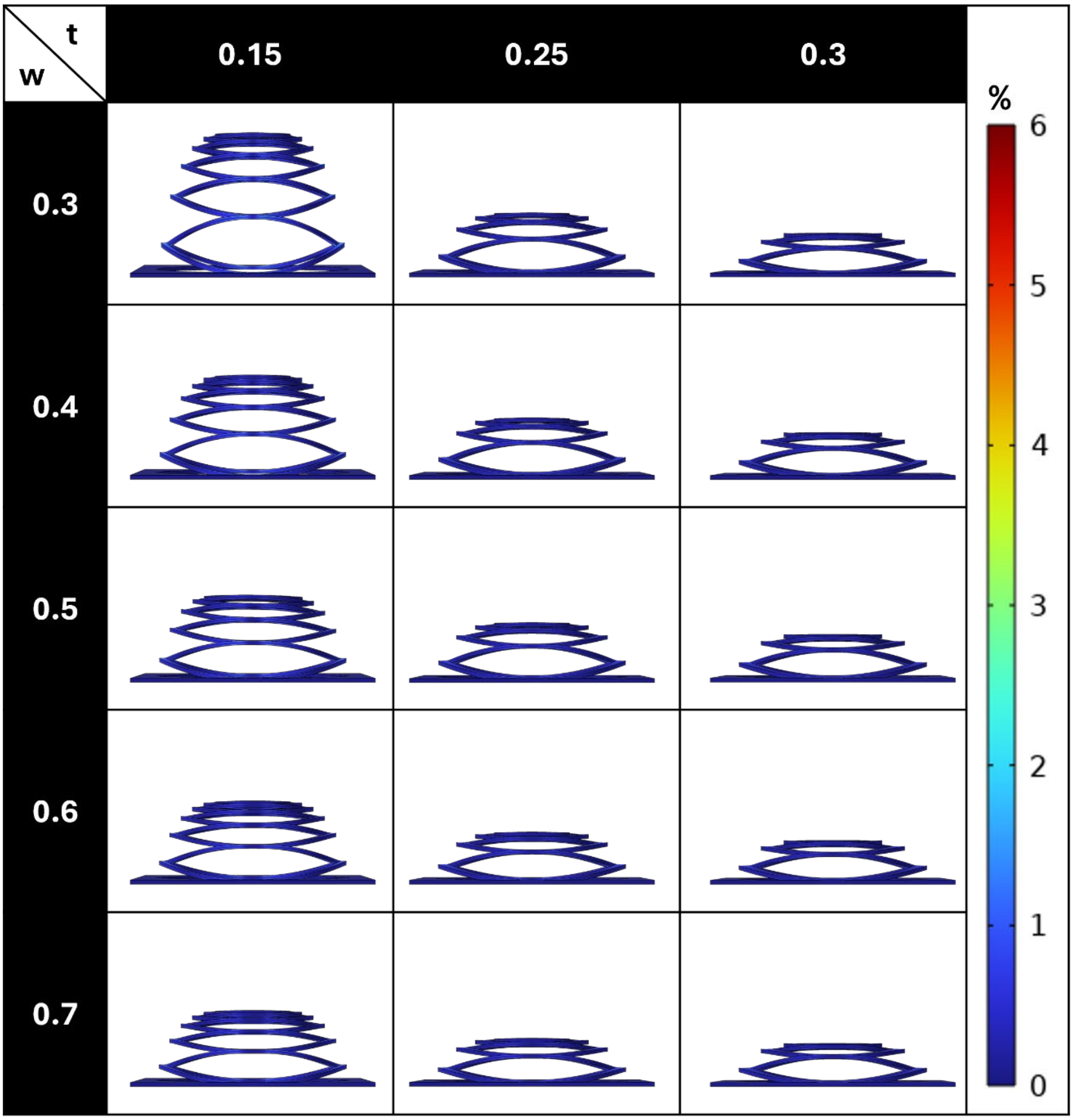

Figure S3. COMSOL-predicted deformation profiles at ~6% maximum principal strain. Kirigami deformation shapes for each (t, w) parameter pair are visualized under maximum principal strain of approximately 6%, forming the simulation dataset used to determine shape-setting pillar heights and validate the geometric relationship between kirigami design and achievable 3D displacement.

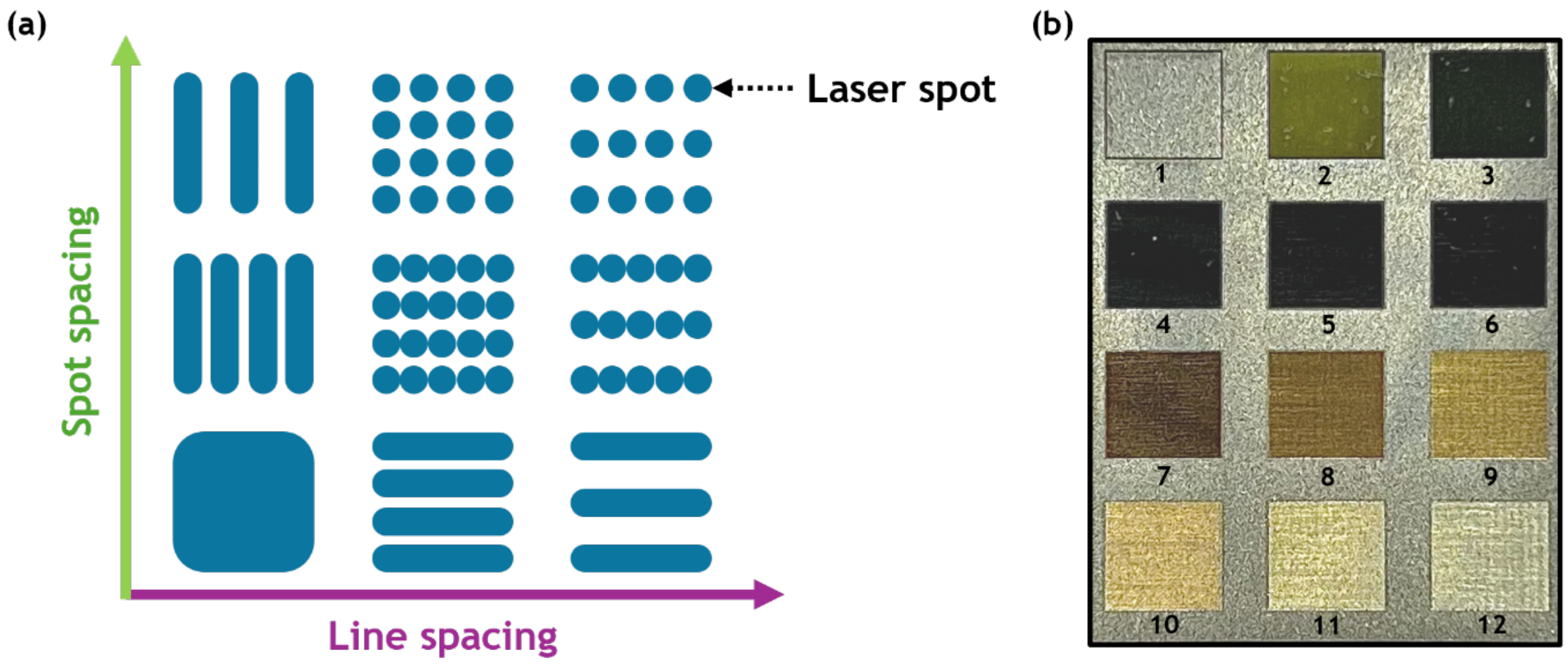

(c)

	1	2	3	4	5	6	7	8	9	10	11	12
Power (%)	Pure	100										
UV Laser Speed (mm/s)		60	120	180	240	300	360	420	480	540	600	660
Pulse Frequency (KHz)		60										
Line Spacing (mm)		0.001	0.002	0.003	0.004	0.005	0.006	0.007	0.008	0.009	0.01	0.011
Z axis focusing (mm)		Laser in-focus (Auto focusing)										

Figure S4. Laser oxidation methodology, SMA surface appearance, and processing parameters. a) Uniform raster-scanning conditions are clarified through schematics defining spot spacing and line spacing. b) laser-processed SMA surfaces at various scan speeds are shown to compare oxidation intensity and morphology. c) Detailed processing table lists power, scan speed, pulse repetition rate, line spacing, and Z-focus values for all oxidation conditions used in optical characterization.

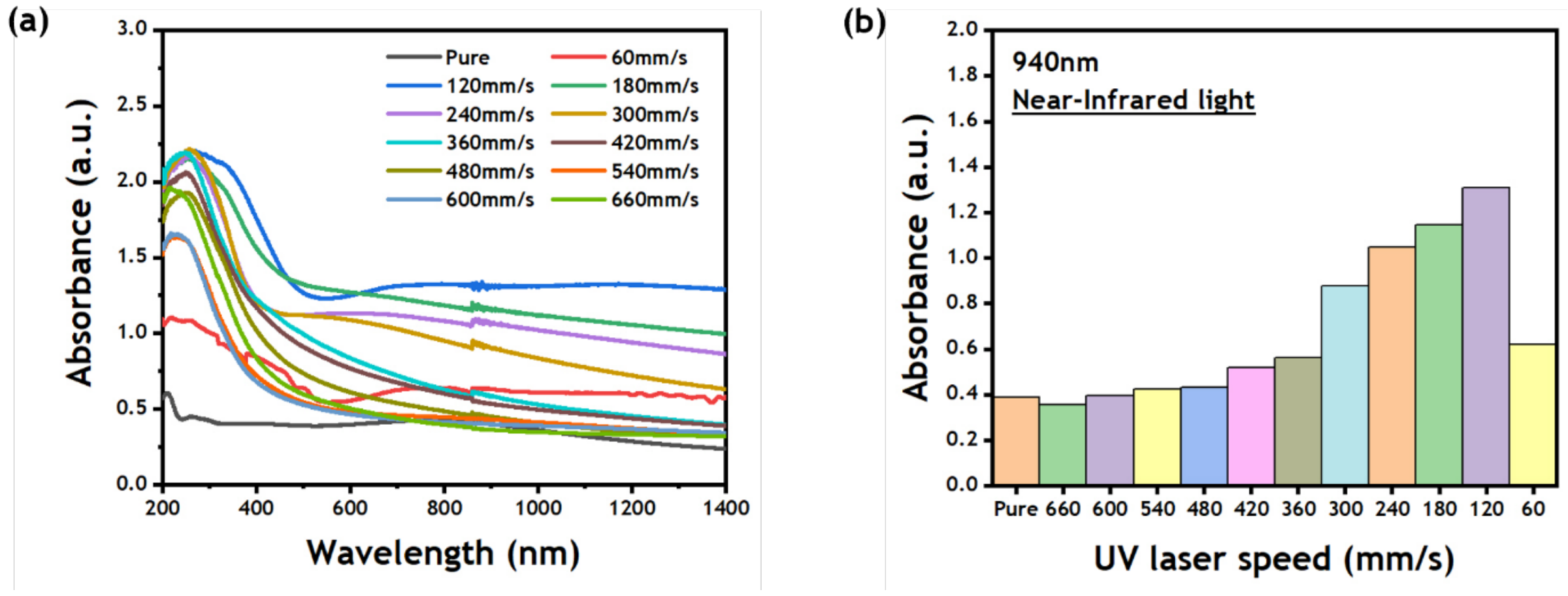

Figure S5. Optical absorbance spectra of laser-oxidized SMA surfaces. a) Full-range absorbance curves (200–1400 nm) for twelve oxidation conditions demonstrate wavelength-dependent optical modification. b) Comparison at 940 nm highlights the variation in NIR absorption relevant to photothermal actuation using the NIR-LED source.

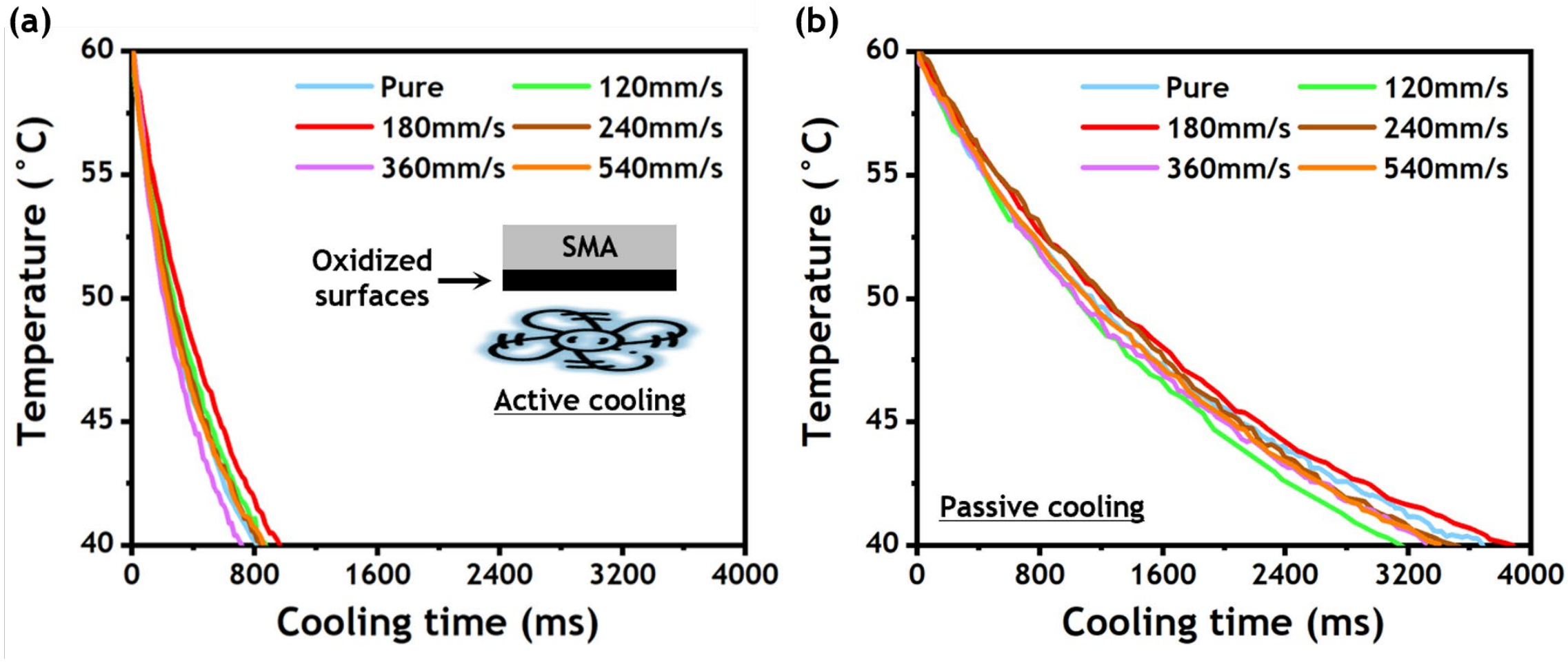

Figure S6. Cooling behavior of UV-laser–oxidized SMA samples. a) Active cooling and b) passive cooling temperature–time curves for pure and oxidized conditions (120–540 mm/s), measured from the same initial temperature (60 °C) for comparison.
